# Epithelioid trophoblastic tumor with lung metastasis: A case report and literature review

**DOI:** 10.1097/MD.0000000000038108

**Published:** 2024-07-05

**Authors:** Jing Li, Zhenwu Du, Tianmin Xu, Chenhong Li, Shumin Ba, He Zhu

**Affiliations:** a Department of Obstetrics and Gynecology, The Second Hospital of Jilin University, Changchun, Jilin, China; b Department of Orthopaedics, The Second Hospital of Jilin University, Changchun, Jilin, China; c Research Center, The Second Hospital of Jilin University, Changchun, Jilin, China.

**Keywords:** epithelioid trophoblastic tumor, intermediate trophoblastic tumors, lung metastatic tumor

## Abstract

**Rationale:**

Epithelioid trophoblastic tumor (ETT) is an extremely rare variant of gestational trophoblastic neoplasms (GTNs). The biological behavior and therapeutic schedule of ETT remains to be defined which frequently poses diagnostic and therapeutic challenges. Although ETT is a relatively indolent malignancy tumor, the therapeutic efficacy and survival rate decrease significantly when presented with metastases. The lung is the most common site of ETT metastasis.

**Patient concerns:**

A 39-year-old female patient presented with irregular vaginal bleeding and slight distention pain in lower abdomen.

**Diagnoses:**

The patient was diagnosed ETT with lung metastasis after surgery and immunohistochemical staining.

**Interventions:**

A total abdominal hysterectomy plus bilateral salpingectomy and histopathology were performed. The patient received 3 cycles of etoposide, methotrexate, actinomycin-D/etoposide, cisplatin (EMA/EP) regimen chemotherapy after surgery. Due to the presence of lung metastasis, she received pulmonary lesion resection and another cycle of postoperative chemotherapy.

**Outcomes:**

The patients showed a good response to treatment initially. However, the patient did not complete the full initial treatment for family reasons and had signs of recurrence after 2.5 months. The serum β-hCG level gradually elevated and the lung imaging showed that the lesion area gradually expanded. After 15 months of follow-up, the patient declined further treatment due to a lack of presenting symptoms.

**Lessons:**

The diagnosis of ETT should be taken into consideration in patients with abnormal vaginal bleeding and low levels of β-hCG. Patients with metastatic disease should be treated with complete surgical resection and intensive combination chemotherapy to maximize the opportunity for cure. Targeted biological agents might be potential therapeutic strategies for chemotherapy-resistant or recurrent patients.

## 1. Introduction

Choriocarcinoma (CC), placental site trophoblastic tumor (PSTT), and epithelioid trophoblastic tumor (ETT) can occur after any type of pregnancy and collectively known as gestational trophoblastic neoplasms (GTNs), and the latter 2 originate from intermediate trophoblastic cells in the placental region, which are commonly called together intermediate trophoblastic tumors (ITTs).^[1]^ ETT is the rarest type of GTNs with only a limited number of case reports.^[2–4]^ The majority of ETTs arise in the lower uterine segment and cervix, followed by the uterine corpus.^[5,6]^ In addition, ETT could also present as a primary uterine disease with metastasis or extra-uterine disease.^[6–10]^ Metastasis from ETT occurred in 25%–35% of cases while lung was the most frequently extrauterine site of ETT.^[10–12]^ It can also be found in the liver, brain, ovarian, vagina and spine, etc.^[13–17]^ Unlike other GTNs, ITTs have a greater propensity for lymphatic spread.^[18]^ Single or multiple sites of metastasis can be present. ETT exhibits similar behaviors and overlapping clinical features to PSTT, including slow growing, relatively low β-hCG concentrations, and less chemotherapy sensitive,^[3,19,20]^ therefore, the treatment approach for both is typically identical. For ITTs, the mainstay of treatment is surgical resection which is different from other GTNs.^[1,3]^ Herein, we report a case of intrauterine ETT with lung metastasis in a 39-year-old premenopausal woman with a systematic review of the literature.

## 2. Case report

**Figure 1.**
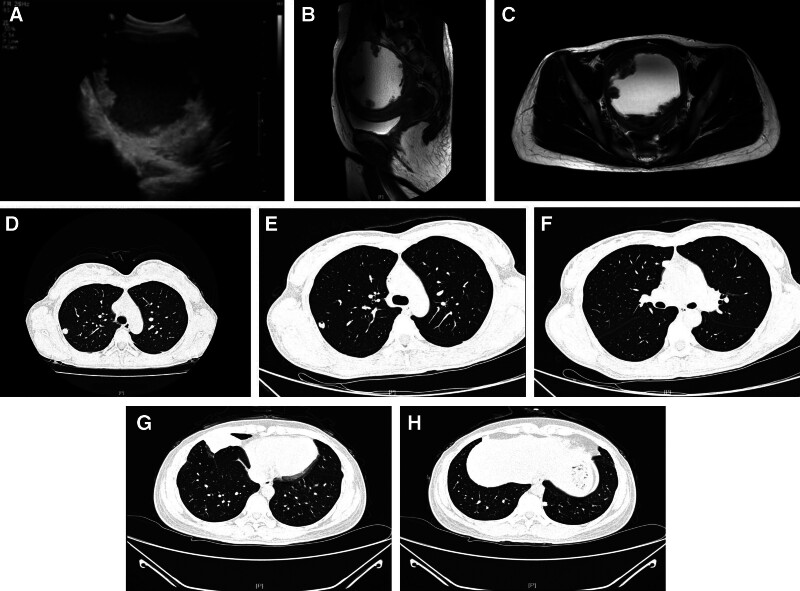
Imaging findings: (A) Ultrasound scan showed a lesion measuring 10.5*10.2 cm with blood flow; (B and C) On T2-weighted images of MRI revealed a 10.4*11.0*10.8 cm mass in the uterine posterior wall, and multiple irregular nodules were observed at the edges and protruded into the lesion; (D) The HRCT scan of the chest on July 23, 2022, showed multiple nodular hyperdensities in both lungs. The largest one about 12 mm*10 mm in size located in the right upper lobe; (E and F) The scan of preoperative HRCT showed multiple nodules in both lungs. A 11 mm nodule in the right upper lobe exhibited an empty bubble within it. (G) The HRCT scan of chest showed multiple soft-tissue densities of both lungs under the pleura which the largest one about 55*34 mm in size located in the subpleural region of right middle lobe. (H) Multiple nodular hyperdensities were revealed in both lungs. The largest one measuring approximately 8mm in diameter located in the left lower lobe. HRCT = high-resolution computed tomography, MRI = magnetic resonance imaging.

**Figure 2.**
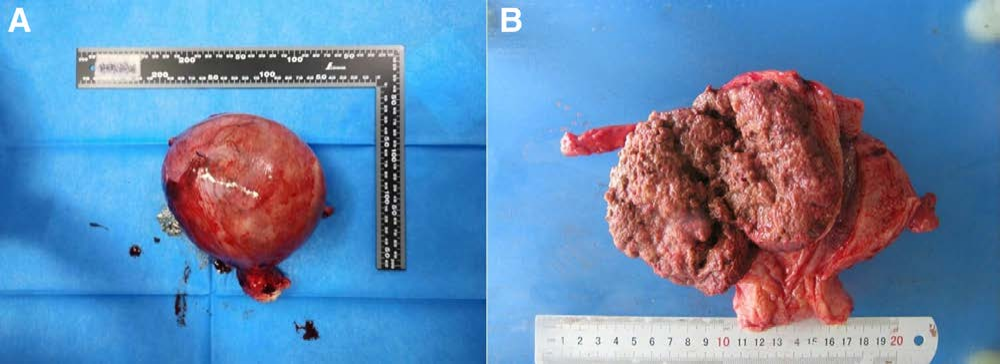
Surgical specimen: (A) The gross view of hysterectomy specimen: (B) The uterus was dissected through an anterior wall incision showing the gray-red, friable mass visible inside.

**Figure 3.**
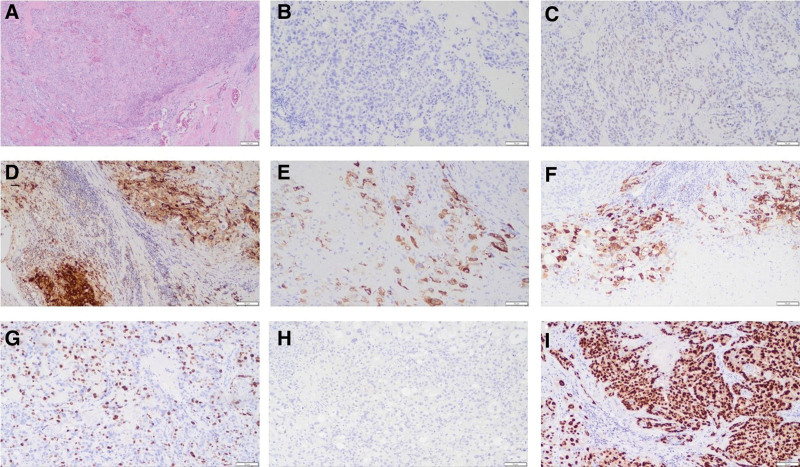
Histological and immunohistochemical features of the uterine tumor: (A) HE staining (×40); (B) immunohistochemical staining for CK (×100); (C) immunohistochemical staining for p63 (×100); (D) immunohistochemical staining for HCG (×100); (E) immunohistochemical staining for hPL (×100); (F) immunohistochemical staining for MUC4 (×100); (G) immunohistochemical staining for Ki67 (×100); (H) immunohistochemical staining for α-inhibin (×100); (I) immunohistochemical staining for GATA3 (×100).

In early July 2022, a 39-year-old, gravida 3, para 1, Chinese woman presented to our hospital with complaints of irregular vaginal bleeding for half a year and slight distention pain in lower abdomen occurred in the past 2 weeks. Ultrasound scan revealed a heterogeneous echo of the posterior wall of the uterus projecting toward the uterine cavity approximately 10.5 × 10.2 cm in size and blood flow signals can be found in periphery (Fig. 1A), which coupled with an elevated level of serum β-hCG (1031.46 mIU/mL). The patient had 1 vaginal delivery 11 years ago, and 2 miscarriages 21months ago and 5 years ago, respectively. An intradermal contraceptive device was implanted in her left upper arm at a private clinic 20 months ago. A magnetic resonance imaging (MRI) scan of the pelvis showed a pelvic tumor (Fig. 1B and C), furthermore, multiple nodular hyperdensities in the right upper and middle lobe were revealed by a high-resolution computed tomography (HRCT) scan of the chest, indicating that might be distant metastasis (Fig. 1D), and there were no definite abnormalities on brain MRI. After 9 days, the blood β-hCG value was 2034.26 mlU/mL which showed an upward trend. Subsequently, the patient underwent exploratory laparotomy with total abdominal hysterectomy and bilateral salpingectomy. During the operation, a 12.0 × 10.0 × 9.0 cm sized spherically enlarged uterus was seen with compression and displacement of surrounding tissues. Gross pathologic examination revealed a 9.5 × 6.0 × 6.0cm, gray-red, friable mass with extensive necrosis within the myometrium (Fig. 2). The boundary between the mass and the myometrium was not clear. Mitotic rate was 17 per 10 high power fields and lymphovascular space invasion was identified. Immunohistochemistry staining indicated that CK(AE1/AE3) (−), P40 (partial +), p63 (local weak +), HCG (local +), hPL (local +), MUC4 (local +), Ki67 (positive rate 40%), Vimentin (−), CD10 (+), α-inhibin (−), SALL-4 (−), SMA (−), GATA3 (+). The histological and immunohistochemical features are presented in Figure 3. Postoperative diagnosis: ETT.

**Figure 4.**
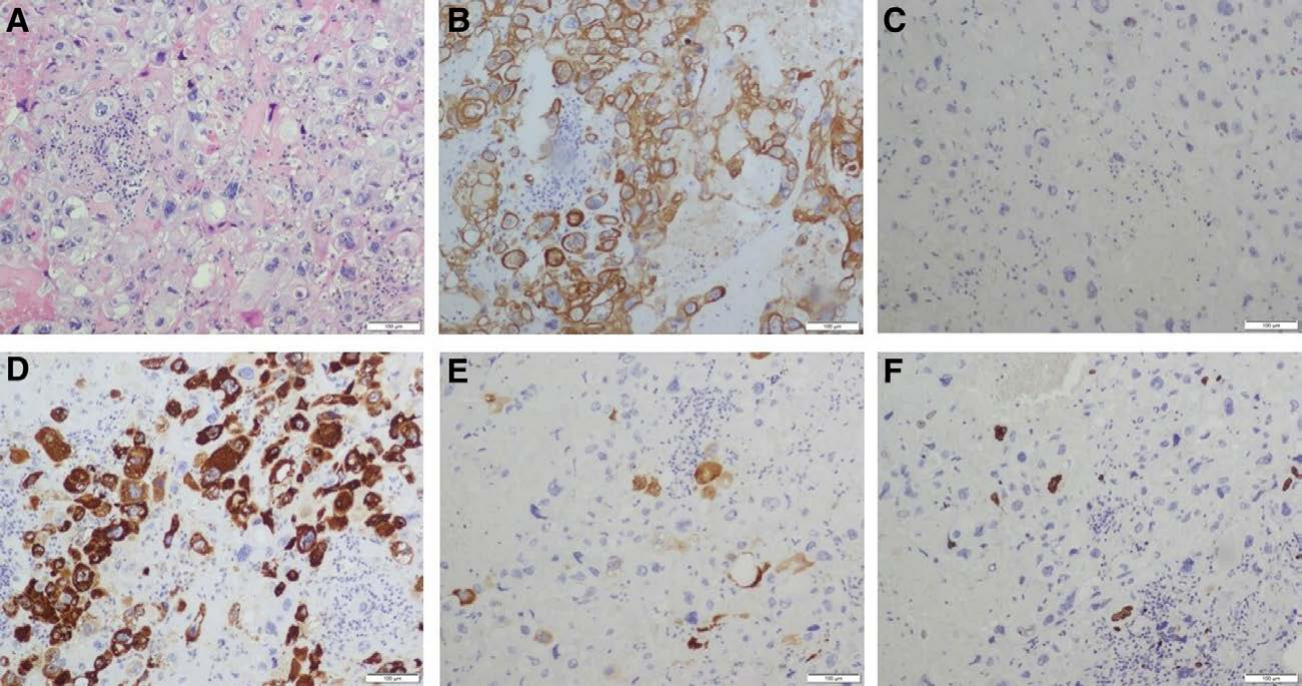
Histological and immunohistochemical features of the lung tumor: (A) HE staining (×100); (B) immunohistochemical staining for CK(AE1/AE3) (×100); (C) immunohistochemical staining for p63 (×100); (D) immunohistochemical staining for hPL (×100); (E) immunohistochemical staining for MUC4 (×100); (F) immunohistochemical staining for Ki67 (×100).

The patient received 3 cycles EMA-EP chemotherapy regimens after the operation, and the levels of β-hCG dropped to normal at the end of the third cycle chemotherapy. Subsequently, the patient transferred to thoracic surgery department for further diagnosis and treatment. A preoperative HRCT scan revealed multiple nodular hyperdensities in the left upper lobe as well as in the right upper and middle lobes. The largest high-density nodule, measuring approximately 11 mm in diameter, was identified in the right upper lobe. Another 11 mm nodule in the right upper lobe exhibited an empty bubble within it (Fig. 1E and F). She underwent the thoracoscopic wedge resection of left upper lobe, right upper and middle lobe. As expected, the histopathologic and immunochemical results were compatible with the diagnosis of ETT. Immunohistochemistry staining indicated that CK(AE1/AE3) (+), p63 (partial +), α-inhibin (focal +), HCG (−), hPL (+), Ki67 (positive rate 10%), TTF-1 (−), Napsin A (−), GATA3 (+), MUC4 (Scattered +), EMA (+), CK8/18 (+), CK5/6 (+). The histological and immunohistochemical features are presented in Figure 4.

**Figure 5.**
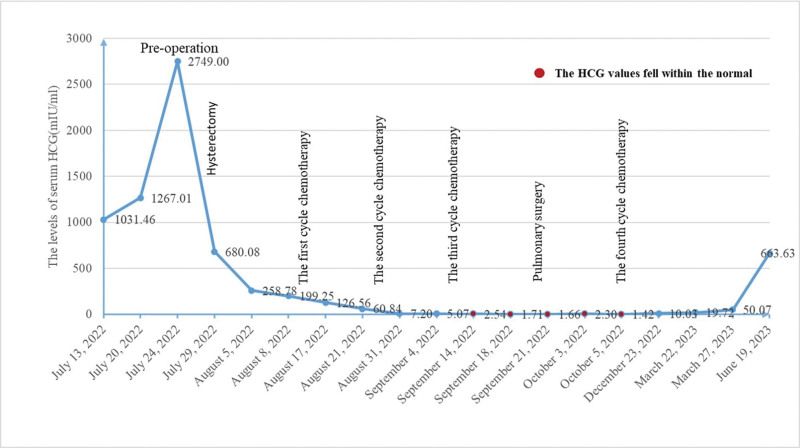
Evolution of serum β-hCG levels over time.

Unfortunately, the patient declined further medical treatment due to family reasons after completing 1 cycle of EMA-EP chemotherapy regimen following lung surgery. An HRCT scan of chest indicated the presence of a pulmonary nodule (6 mm) before the fourth cycle chemotherapy regimen, whereas the follow-up examination conducted 2 months after the completion of chemotherapy did not reveal any definitive nodular lesion. There was irregular monitoring of the levels of serum β-hCG after discharge. During follow-up, serum β-hCG increased to 10.03 mIU/mL about 2.5 months after the last cycle of chemotherapy regimen. The patient declined the recommendation to continue treatment. The blood β-hCG value rose to 663.63 mIU/mL until 8 months after the last chemotherapy regimen. The changes in β-hCG levels are shown in Figure 5. Eleven months after the last chemotherapy regimen, the HRCT scan of chest showed multiple soft-tissue densities of both lungs under the pleura which the largest one measuring approximately 55*34 mm in size (Fig. 1G). And multiple nodular hyperdensities were revealed in both lungs which the largest one about 8 mm in diameter located in the left lower lobe (Fig. 1H). She was not a surgical candidate due to extensive metastases in her lungs. The patient remained asymptomatic at the time of reporting, and we are continuing to encourage the patient to proceed with treatment.

## 3. Discussion

ETT is an extremely rare form of GTN which arises from the chorionic-type intermediate trophoblast. It was previously reported that tumors contain unique paternal genomic element which indicated ETT was associated with prior gestational events.^[21]^ ETT could secondary to full-term pregnancy, hydatidiform mole, ectopic pregnancy, and GTN; however, there have been also reported that nulliparous patients diagnosed ETT, likely with an undocumented spontaneous abortion.^[3,22]^ ETT mostly presents in reproductive-aged women, but there have been reported cases of postmenopausal patients.^[23,24]^ And the occurrence of ETT in male patients is particularly rare, only about 7 cases have been reported in the English literature to data.^[25,26]^ The reported interval between the antecedent pregnancy and the clinical manifestation ranges from 1 week to decades.^[16,27]^

ETT has no specific symptom or sign and tends to be diagnosed by pathological assessment. Patients with ETT performed different clinical presentations which depending on the site of involvement. The majority of patients underwent abnormal vaginal bleeding, and others complained amenorrhea, abdominal pain, and abdominal bloating.^[3,6]^ And dyspnea or hemoptysis may be found due to lung involvement. However, it is sometimes entirely asymptomatic at the time of diagnosis, especially in patients who have extra-uterine ETT.^[3,28]^ Unlike in patients with CC (>10,000 IU/L), the levels of serum β-hCG generally mildly elevated in patients with ETT (<2500 IU/L). Interestingly, the levels of β-hCG is persistent negative in some patients and markedly elevated β-hCG levels (>10,000 IU/L) in patients with mixed GTN which indicated β-hCG levels may not be reliable for the diagnosis and follow-up of ETT, nevertheless, β-hCG levels may be higher in advanced stage disease.^[11,29]^ The clinical manifestations, imaging, and β-hCG levels are easily misdiagnosed as ectopic pregnancy and CC.^[3,29,30]^

Unlike CC which can be diagnosed based on clinical presentation and significantly elevated serum levels of β-hCG, ETT and PSTT are mainly diagnosed based on specific pathological evidence. Given that different treatments are recommended for each tumor, it is critical to distinguish ETT from other tumors. The diagnosis of ETT is easily delayed due to nonspecific clinical features. ETT may be confused with squamous cell carcinoma (SCC) because of its frequent involvement of the lower uterine segment, the appearance of epithelioid histologic morphology, positivity for p63 and keratins, and its eosinophilic hyaline material similar to keratin.^[31]^ The expression of P16 is helpful to differentiate ETT and SCC, as the latter is positive whereas ETT is negative.^[32]^ Furthermore, Ki-67 proliferative index is always high (>50%) in SCC which is usually >10% in ETT.^[1,33]^ DNA fingerprinting analysis demonstrating chimerism is a powerful diagnostic application in cases with equivocal immunohistochemistry.^[34]^ HPV in situ hybridization may help in making the diagnosis of HPV-related cervical lesions. In clinical work, ETT may also be misdiagnosed as CC or PSTT.^[35]^ P63 plays an essential role in differential diagnosis of ETT and PSTT, which is positive in ETT and negative in PSTT.^[36]^ In the differentiation from CC, ETT component shows a relatively uniform population of mononuclear intermediate trophoblastic cells, however, CC is composed of a typically biphasic pattern with multinuclear syncytiotrophoblast and mononuclear cytotrophoblast.^[29]^ CC specifically expressed SALL4 which may be helpful in the differential diagnosis.^[37]^ Moreover, HCG is focally positive in ETT but is diffusely positive in CC, and the Ki-67 proliferative index is a useful tool to distinguish ETT from CC.^[19]^ However, ETT could coexist with PSTT and CC which makes the diagnosis and treatment more difficult.^[35,38]^ The coexistence of CC should be considered in ITTs patients with high β-hCG levels.^[29]^

ETT is a relatively indolent malignancy tumor which the disease survival is more than 90% when there are no metastases, but it drops to 50% to 60% when there are metastases.^[39,40]^ The treatment of ETT is predominately based on the stage of disease with some appropriate consideration of high-risk factors. ETT is more chemo-resistant compared to CC, the primary therapeutic option of ETT is complete surgical removal. Thus, total hysterectomy for early-stage disease and metastatic or residual foci resection in metastatic disease were critical to achieve remission and avoid relapsed disease.^[3,12,29,41–43]^ Sugrue et al reported the overall survival is similar regardless of minimally invasive or open abdominal hysterectomy.^[44]^ For premenopausal women, it is not considered to conventional removal of the ovaries.^[30,41]^ Lymphadenectomy can be performed in patients, whereas it is uncertain if this improves survival.^[11,45]^ If preoperative radiological lymph node involvement is suspected, lymph node sampling is recommended due to the potential for ETT to spread through lymphatics.^[45,46]^ Surgical treatment combined with multiple-drug chemotherapy, immunotherapy and targeted therapy should be considered during the late stage of ETT.

The International Federation of Gynecology and Obstetrics (FIGO) system could be used to stage ETT, whereas the prognostic score is not applicable.^[47]^ Due to the characteristic of more chemo-resistant for ETT, single-agent chemotherapy is insufficient and multi-agent treatment is required for poor prognosis and/or metastatic disease.^[2,48]^ Given the rarity of the disease and the lack of controlled trails, the optimal chemotherapy regimen for ETT remains to be defined. But the investigators reported that platinum-based and high-dose chemotherapy may useful to improve survival in poor prognosis patients.^[30,48–50]^ Therefore, platinum-based regimens such as EMA/EP or paclitaxel, cisplatin/paclitaxel, etoposide are recommended chemotherapy for patients with ETT, especially those with persistently positive levels of β-hCG after surgery, metastatic lesions and are inoperable.^[11,30,42,48,50]^ There is controversy about whether patients in stage I disease benefit from adjuvant chemotherapy. Patients with normal levels of β-hCG in stage I seem to be enough to receive surgery alone.^[3,30,51]^ Nevertheless, it is still uncertain. Adjuvant treatment should be considered in cases such as stage I with poor prognostic factors and indeed, stage II to IV.^[1,39,45,52]^ It has been reported that time interval > 48 months between antecedent pregnancy, FIGO stage, mitotic index of tumor cells >5 to 10 high power fields, deep myometrial invasion, necrosis, the presence of metastases as the possible poor prognostic factors of ETT.^[3,10,11,49,53–57]^ Besides, Yang et al suggested that the positive expression of LAG-3 was a prognostic factor for disease recurrence.^[58]^ But the timing, type and duration of multi-agent chemotherapy are unclear. Zhang et al reported that serum β-hCG is not the only factor of chemotherapy withdrawal and follow-up monitoring, and imaging examination are more essential for ETT.^[54]^ Additionally, different approaches to the sequencing of surgery and chemotherapy have been used, although the best approach is not clear. Here, we summarized the characteristics of uterine primary ETT with metastasis or extrauterine ETT in case reports, as shown in Table 1. In our case, the patient was treated with total abdominal hysterectomy plus bilateral salpingectomy. She received 3 cycles of EMA/EP regimen chemotherapy after surgery and the serum levels of β-hCG returned to normal. Due to the presence of lung metastasis, she received pulmonary lesion resection and 1 cycle of postoperative chemotherapy. The patients showed a good response to treatment initially. However, the initial planned 4 to 5 cycles of chemotherapy following the lung surgery were not completed due to the patient’s noncompliance. Unfortunately, the disease recurred rapidly after treatment interruption. Despite our repeated attempts to encourage the patient to receive treatment upon the recurrence of the disease, the latter still refused because of the absence of symptoms. Therefore, we failed to assess the final outcomes of this treatment regimen for lung metastases in the ETT patient.

**Table 1:** Overview of the English literature on uterine primary epithelioid trophoblastic tumor with metastasis or extrauterine epithelioid trophoblastic tumor.

Case	Uterus	Metastatic/Extrauterine sites	Age	AP/interval	Symptoms	hCG (mIU/mL)	Surgical treatment	Histology	Chemotherapy(courses)	Relapse/ metastasis	Outcome	FU
Fenichel et al^[59]^	−	Lung	29	FTD**/**4 yr	VB	low levels	1. Left ovariectomy; 2. Lobectomy of the lung	ETT	1. After the second surgery, EP-EMA (6)	/	NED	12 mo
Imamura et al^[38]^	+	Lung	32	FTD**/**2 yr	Amenorrhea	93,820	1. Excision of the Uterine horn mass	ETT&CC	1. After the surgery, MEA (6)	/	NED	26 mo
Li et al^[60]^	+	Lung	46	FTD**/**10 yr	VB	4396	1. Hysterectomy; 2. Lobectomy of the lung	ETT	1. EMA-CO (5); 2. EMA-EP (5)	/	NED	5 mo
Li et al^[60]^	+	Lung	31	Abortion**/**3 yr	VB	46.6	1. TLH; 2. Lobectomy of the lung	ETT	1. EMA-CO (5); 2. BEP (4)	/	Death	16 mo
Li et al^[60]^	+	Lung, pancreas	44	Abortion**/**8 mo	VB	17,4315.5	1. Hysterectomy	ETT	1. EMA-CO (8)	/	PFS	30 mo
Shih et al^[19]^	**/**	Lung	42	FTD**/**ND	Lung mass	1300	1. Lobectomy of the lung	ETT&CC	1. After the surgery, actinomycin-D, VP16, methotrexate and leucovorin (unknown)	/	Lost	**/**
Shih et al^[19]^	**/**	Bowel, lung	39	FTD**/**ND	Bowel obstruction	60	1. Bowel resection; 2. Lobectomy of the lung	ETT	1. After the first surgery, vincristine, methotrexate, and Cytoxan (unknown)	/	Death	36 mo
Black et al^[61]^	/	Lung	47	HM/>4 yr	Cough	24,494	1. Lobectomy of the lung	ETT	1. Prior to the surgery, EMA-CO (10), relapsed after chemotherapy 2 mo	/	NED	34 mo
Urabe et al^[62]^	/	Lung	38	FTD/ND (HM/11 yr)	Poor physical condition	80.1	1. A partial resection of the lung tumor; 2. Lobectomy of the lung	ETT	1. After the first surgery, EMA-CO (6)	/	hCG (+)	3 mo
Niu et al^[63]^	/	Lung	26	FTD/1.5 yr	Home pregnancy test positive	350	1. Lobectomy of the lung.	ETT	/	/	hCG (−)	1w
Knox et al^[64]^	+	Lung	20	FTD/7 mo	VB	106,153	1. TAH	ETT&CC	1. Prior to the surgery, EMA-CO (5); 2. After the surgery, EMA-CO (3); 3. Relapsed within 4 mo, EP-EMA (2) + HDC/ASCR (cyclophosphamide, etoposide, carboplatin)	/	NED	27 mo
Ahn et al^[65]^	/	Lung	26	Noncertified	Menstrual delay	11.37	1. Lobectomy of the lung and mediastinal nodal dissection	ETT	1. After the first Surgery, EMA-CO (6)	/	NED	9 mo
Okereke et al^[66]^	/	Lung	40	ND	VB	1100	1. Lobectomy of the lung and mediastinal nodal dissection	ETT	1. After the first Surgery, PE (4)	/	NED	12 mo
Abrao et al^[67]^	−	Lung	31	ND/8 yr	VB	700	1. Lobectomy of the lung	ETT	−	−	NED	12 mo
Kim et al^68]^	−	Lung	35	ND	Abdominal pain	Normal	1. A biopsy of the lung mass; 2. Lobectomy of the lung	ETT	−	−	NED	15 mo
Li et al^[69]^	−	Lung	31	Noncertified/2 yr	Low levels of HCG > 2 yy	168.1	1. D&C 2. Lobectomy of the lung	ETT	1. After the surgery, EP-EMA (3)	−	NED	13 mo
Lewin et al^[28]^	−	Lung	38	FTD/42 mo	ND	400	1. D&C; 2. Wedge resection, lobectomy of the lung; 3. TAH&USO	ETT&CC	−	−	NED	90 mo
Lewin et al^[28]^	−	Lung	49	Abortion/12 mo	VB	2204	1. D&C; 2. A biopsy of the lung mass; 3. Lobectomy of the lung, mediastinal nodal dissection; 4. LAVH&BSO	ETT	1. After D&C MTX (2); 2. After the biopsy, EMA-EP (3.5)	−	NED	45 mo
Lewin et al^[28]^	−	Lung	34	FTD/24 mo	Irregular menses	426	1. Laparoscopy and D&C; 2. Segmentectomy	ETT	1. After the first surgery, Methotrexate, leucovorin (4); 2. After the second surgery, EMA-EP (3)	−	NED	22 mo
Tsai et al^[18]^	+	Lung, Pelvic lymph node	20	FTD/1 yr	VB, dyspnea	2229	1. mRAH&BPLND	ETT&PSN	1. Prior to the surgery, EMA-CO (5); 2. After the surgery, EMA-CO (2)	−	NED	47 mo
Prabha Devi et al^[5]^	+	Lung, brain	26	Abortion/6 mo	VB	68,000	1. TAH	ETT	1. After the surgery, EMA-CO (12)	/	Remission	9 mo
Fadare et al^[70]^	+	Lung, peritoneum	29	FTD/3 yr	ND	2400	1. D&C and peritoneal biopsy; 2. Hysterectomy	ETT	ND	+	Death	8 mo
Fadare et al^[70]^	+	Lung, pelvic	42	ND/13 yr	Amenorrhea	200	1. The biopsies of bladder wall and vesicouterine fold; 2. The biopsies of peritoneum and abscess wall; 3. Radical hysterectomy, BS and lymph node dissection	ETT	1. After the second surgery, EMA-CO (3); 2. After the last surgery, EMA-CO (3)	+	Metastasis	1 mo
Jashnani et al^[71]^	/	Lung, spleen	24	Abortion/2 mo	Menorrhagia, dyspnea	300,000	1. Autopsy	ETT (lung, spleen) & CC (Uterus, vagina, ovarian)	1. Antitubercular therapy	−	Death	−
Kuo et al^[72]^	−	Broad ligament	41	Abortion/9 yr	VB	126,763	1.TAH&BSO; 2. Broad ligament mass resection	ETT	1. Diagnosed GTN without tissue proof 9 yr ago, MTX (15); 2. EMA (9)	−	NED	24 mo
Gallardo et al^[31]^	−	Broad ligament	41	FTD**/**10 yr	Abdominal pain	900	1. BS; 2. Hysterectomy and broad ligament mass resection	ETT	−	−	NED	ND
Zeng et al^[73]^	/	Cesarean scar	39	FTD/3 yr	Abdominal mass	Normal	1. Abdominal mass excision, endometrial curettage; 2. Relapsed: The second time abdominal mass resection; 3. Relapsed: TAH&BS, the third time abdominal mass resection	ETT&CC	1. After the first Surgery, EP (5)	+	NED	ND
Hsiue et al^[74]^	−	Abdominal wall	54	Abortion/23 yr	Abdominal mass around Cesarean section scar	8.36	1. Two incisional biopsies; 2. Wide excision of the tumor; 3. Relapsed after 6 mo: excision of the tumor and multiple intestinal metastases		1. Prior to the surgery, EMA-CO (3) 2. Relapsed after the last surgery 2 mo, ifosfamide and etoposide	+	Relapse	8 mo
Yang et al^[75]^	−	Abdominal wall cesarean scar	39	FTD**/**3 yr	anterior abdominal wall mass	<1.2	1. local abdominal wall lesion excision twice; 2. SAH-BS, left ovarian cyst resection	ETT&CC	1. Prior to the first surgery, EP (2)	−	NED	12 mo
Niu et al^[63]^	−	Anterior abdominal wall, omentum, intestine	61	ND	VB	ND	1. Exploratory laparotomy, Omentectomy	ETT	−	−	Hospice care	ND
Khunamornpong et al^[76]^	−	Ovarian	32	ND (HM/5 yr)	Palpable pelvic mass	ND	1.USO	ETT	1.After the surgery, EMA (5); 2.Relapsed after 6 mo, Cisplatin/ifosfamide (6)	+	NED	19 mo
Wang et al^[33]^	−	Ovarian, fallopian tube	29	FTD/20 mo	VB	12,878	1. Oophorectomy (left), salpingectomy and D&C	ETT	ND	−	NED	ND
Park et al^[77]^	−	Ovarian, lung	75	ND/47 yr	Lung mass	57,971	1. TAH&BSO	ETT	1. After the surgery, EMA-CO (5)	−	PFS	5 mo
Mahmood et al^[78]^	−	Ovarian, lung, liver	43	FTD/10 yr	VB, amenorrhea	3265	1. TAH&BSO	ETT	1. Clear cell carcinoma of the ovary was diagnosed initially, TC (3)	+	Death	−
Arafah et al^[8]^	−	Ovarian, peritoneal, liver and lung	50	FTD/>10 yr	Abdominal pain and distension	806.7	1. The biopsy of the ovarian mass	ETT	1. After the biopsy, EMA-EP	−	−	−
Ohira et al^[15]^	+	Vagina	30	FTD/15 mo	Vaginal tumor and pain	2.8	1. Local excision of the vaginal tumor; 2. Hysteroscopy target biopsy and endometrial curettage; 3. TAH and wide local excision of the vagina	ETT	−	−	NED	14 mo
Zhao et al^[79]^	−	Vagina	43	Abortion/3.5 yr	Soft-tissue block vagina	ND	1. Cystectomy; 2. Relapsed after 5 mo: Vaginal lesion resection	ETT	1. Prior to second surgery, FAEV (3) 2. After the surgery, FAEV (1)	+	NED	ND
Keser et al^80]^	+	Vaginal cuff (Recurrence 4 yr) Lung (After 5 yr)	47	ND**/**16 yr	Abdominal pain and distension	5.09	1. TAH-BSO	ETT	−	+	Relapse and metastasis	5 yr
Helbig et al^[13]^	+	Liver	46	ND**/**8 yr	VB	142	1. Excision of liver metastases; 2.TAH&BSO; right pelvic lymphonodectomy; 3. Thermoablation of the liver	ETT	1. Prior to the first surgery and after the second surgery, EMA-EP (8); 2. Pembrolizumab (24)	−	NED	48 mo
Chohan et al^[14]^	−	Spine, liver, lung	36	FTD**/**2 wk	Low back pain	Unknown	1.A thoracic T10 corpectomy with a T9 and T10 laminectomy.	ETT	1. After the surgery, EMA-CO (unknown)	−	Death	9 mo
Theodossiadis et al^[81]^	−	Choroid, skin, liver	37	FTD/16 mo	Visual field defect	120	1. Laparotomy; 2. A biopsy of skin lesion	ETT	After the surgery, etoposide, methotrexate, actinomycin-D, cyclophosphamide, vincristine, and cisplatin (unknown)	−	NED	12 mo
Macdonald et al^[82]^	−	Gallbladder, liver	41	FTD/6 yr	VB	14425	1. The biopsy of the liver	ETT	1. CEC (6); 2. TC; 3. EMA-CO	+	Death	7 mo
Macdonald et al^[82]^	+	liver, pancreas, kidneys, lung, spine	59	FTD/30 yr	A painless enlargement of a right cervical lymph node and back pain	148,460	1. TAH-BSO (10 yr ago); 2. The biopsy of the cervical lymph node	ETT	After the biopsy: 1.EP-EMA (8); 2.TC; 3.MICE; 4. Intramuscular methotrexate	+	Death	27 mo
Nakamura et al^[16]^	+	Brain, liver, lung	32	FTD/1 wk	Abdominal pain	5038	1. Embolization of the liver hematoma; 2. Liver biopsy; 3. Stereotactic brain radiation; 4. Hysterectomy and biopsy of liver hematoma	ETT	1. After liver biopsy, EP (2); 2. Combination of brain radiation, HD EMA-EP (2); 3. After treatment of brain metastases and prior to hysterectomy, EMA-EP (3)	/	NED	12 mo
Madhu et al^[83]^	/	Omentum, peritoneal, liver	45	None	Vomiting, diarrhea, abdominal distention	443	**−**	ETT	1. EMA-CO (1)	/	Death	20 d
Bell et al^[84]^	**/**	thorax, intracardiac, abdominal soft tissues	47	ND**/**12 yr	recurrent respiratory infections	9	**−**	ETT	1. EMA/EP (7); 2. Pembrolizumab (29 and continue treatment)	/	PFS	16 mo
Aiob et al^[85]^	+	Bladder (After 24 mo)	49	FTD/11 yr	Menstrual delay	887	1. TAH-BSO, pelvic node sampling; 2. A biopsy of bladder wall (After TAH-BSO 24 mo)	ETT &PSTT	−	+	Relapse (ETT)	24 mo
Basto et al^[9]^	−	Vulva, lung, kidney, ganglia	40	ND	Multiple skin nodules	556	1. A biopsy of vulvar lesion	ETT	1. EMA-CO (3)	−	Death	2 mo
Tse et al^[86]^	+	Ureter (After 72 mo)	32	Abortion/<12 mo	Abdominal pain	ND	1. TAH; 2. Right ureteric resection and reimplantation	ETT	−	−	NED	88 mo
Jiang et al^[87]^	−	Recto-uterine pouch	30	FTD/5 yr	VB	577.05	1. Tumor resection	ETT	1. After the surgery, EMA-CO (3)	−	NED	ND
Gil et al^[88]^	+	Skin, vulva, intestine, pleura, et al (systemic metastases)	40	FTD/9 yr	VB, cough, pain	1187	1. The biopsy of skin and vulvar nodule; 2. Endoscopic studies; 3. Palliative radiation therapy	ETT	1. Stopped due to medical complications, EMA-CO	−	Death	7 mo
Parker et al^[89]^	/	Fallopian tube	39	Abortion/2 yr	Pelvic pain	52,000,000	1. TAH-USO, infracolic omentectomy	ETT	1. After the surgery, EMA-CO (6)	−	NED	12 mo
Noh et al^[90]^	/	Paracervix, parametrium, peri adnexal soft tissue	44	ND	Abdominal pain and distension	/	1. TLH-BSO, ascites cytology, peritoneal biopsies; 2. Cytology, laparoscopic pelvic peritonectomy, partial; omentectomy, appendectomy, and PLNS	ETT	1. After the first surgery, EMA (4)	−	NED	12 mo

− = none, & = and, + = yes, AP = antecedent pregnancy, BEP = bleomycin, etoposide, cisplatin, BPLND = bilateral pelvic lymph node dissection, BS = bilateral salpingectomy, BSO = bilateral salpingo-oophorectomy, CC = choriocarcinoma, CEC = cyclophosphamide, etoposide, and cisplatin, d = day, D&C = diagnostic curettage, EMA = etoposide, methotrexate, actinomycin-D, EMA/CO = etoposide, methotrexate, actinomycin-D/cyclophosphamide, vincristine, EMA/EP = etoposide, methotrexate, actinomycin-D/etoposide, cisplatin, EP = etoposide, cisplatin, EP/EMA = etoposide, cisplatin/etoposide, methotrexate, actinomycin-D, ETT = epithelioid trophoblastic tumor, FAEV = floxuridine,dactinomycin, etoposide, vincristine, FTD = full-term delivery, FU = follow-up, GTN = gestational trophoblastic neoplasms, HD = high-dose, HDC/ASCR = high-dose chemotherapy regimen with autologous stem cell rescue, HM = hydatidiform mole, LAVH = laparoscopic-assisted vaginal hysterectomy, m = month, MEA = methotrexate, etoposide. actinomycin-D, MICE = mesna, isosfamide, carboplatin, etoposide, mRAH = modified radical hysterectomy, MTX = methotrexate, ND = not described, NED = no evidence of disease, PE = cisplatin, etoposide, PFS = Progression-free survival, PLNS = pelvic lymph node sampling, PSN = placental site nodule, SAH = subtotal abdominal hysterectomy, TAH = total abdominal hysterectomy, TC = taxol, carboplatin, TLH = total laparoscopic hysterectomy, USO = unilateral salpingo-oophorectomy, VB = vaginal bleeding, w = week, y = year.

The use of high-dose chemotherapy (HDC) with peripheral blood stem cell support appears to be active in patients with refractory and relapsed disease.^[64,91,92]^ In the study by Frijstein et al, 45% (5/11) of patients with ETT/PSTT presenting ≥ 48 months after antecedent pregnancy were in remission.^[91]^ But they also suggested that it should consider the risk of life-threatening complications and the emergence of new less toxic salvage therapies such as pembrolizumab before using HDC.^[91,93]^ Since a significant amount of gestational trophoblastic tumors, including ETT, express the PD-L1 receptor, there has been reported immunotherapy using pembrolizumab as a successful treatment in ETT.^[13,42,52,58,84]^ Pembrolizumab has been hypothesized to be a reasonable option for therapy of ETT patients with significant PD-L1 positivity and especially chemo-resistance or recurrent. Additionally, EGFR, VEGF, PD-L2, B7-H3, VISTA, LPCAT1, and CD105 were identified as potential therapeutic targets for metastatic or recurrent ETT.^[10,42]^

Data on fertility-sparing therapy in ETT are even more scarce. There have been reported 4 patients only received lesion excision with or without chemotherapy, no signs of recurrence were found during the followup period.^[38,86,94]^ Conversely, Davis^[3]^ and Liu et al^[49]^ reported 3 patients with ETT refused hysterectomy for their desire of ongoing fertility who subsequently developed recurrence or progressive disease. The delay in instigating surgical treatment may account for the recurrence and death. Given these discrepant results, the safety of fertility-sparing treatment in ETT remains uncertain. For young women who strongly desire to preserve their childbearing potential, a fertility-sparing approach should be only considered for accurately selected patients after an adequate and extensive counseling. It is not suitable to preserve fertility in patients with diffuse lesions.^[1]^

## 4. Conclusion

ETT is a rare potentially high-risk GTN with the variability in presentation which may lead to delay in treatment. Surgery is the mainstay of treatment in ETT. For metastatic disease and localized disease with poor prognostic factors, combined surgical resection and muti-chemotherapy are recommended to maximize the opportunity for cure. However, many questions remain to be resolved regarding the optimal management of ETT. Further studies on the exploration of potentially valuable therapeutic strategies are urgently needed. More cases and data of ETT should be reported to implement the knowledge on ETT management and shape future therapies.

## Acknowledgments

We have obtained a signed consent form from the patient and her guardian and this form will be filed with my records. We would like to thank the patient for their participation in this study.

## Author contributions

**Conceptualization:** Tianmin Xu.

**Data curation:** Shumin Ba.

**Formal analysis:** Jing Li.

**Investigation:** Chenhong Li.

**Software:** Zhenwu Du.

**Writing – original draft:** Jing Li.

**Writing – review & editing:** Jing Li, He Zhu.

## References

[1] Ngan HYS Seckl MJ Berkowitz RS. Diagnosis and management of gestational trophoblastic disease: 2021 update. Int J Gynaecol Obstet. 2021;155 Suppl 1(Suppl 1):86–93. 10.1002/ijgo.13877 PMC9298230 34669197

[2] Froeling FE Seckl MJ. Gestational trophoblastic tumours: an update for 2014. Curr Oncol Rep. 2014;16:408. 25318458 10.1007/s11912-014-0408-y

[3] Davis MR Howitt BE Quade BJ. Epithelioid trophoblastic tumor: a single institution case series at the New England Trophoblastic Disease Center. Gynecol Oncol. 2015;137:456–61. 25773203 10.1016/j.ygyno.2015.03.006

[4] Zhao S Sebire NJ Kaur B. Molecular genotyping of placental site and epithelioid trophoblastic tumours; female predominance. Gynecol Oncol. 2016;142:501–7. 27246306 10.1016/j.ygyno.2016.05.033

[5] Prabha Devi K Bindhu Priya N Himabindu P. Metastatic epithelioid trophoblastic tumor: a rare case report. J Obstet Gynaecol India. 2014;64:212–4. 24966508 10.1007/s13224-012-0261-6 PMC4061333

[6] Kim JH Lee SK Hwang SH. Extrauterine epithelioid trophoblastic tumor in hysterectomized woman. Obstet Gynecol Sci. 2017;60:124–8. 28217684 10.5468/ogs.2017.60.1.124 PMC5313356

[7] Lei W Zhang F Zheng C. Metastatic epithelioid trophoblastic tumor of the lung: a case report. Medicine (Baltimore). 2018;97:e0306. 29668580 10.1097/MD.0000000000010306 PMC5916664

[8] Arafah MA Tulbah AM Al-Husaini H. Extrauterine epithelioid trophoblastic tumor arising in the ovary with multiple metastases: a case report. Int J Surg Pathol. 2015;23:339–44. 25695493 10.1177/1066896915570661

[9] Basto R Brandao Rego I Correia Magalhaes J. Epithelioid trophoblastic tumour presenting with scalp lesions. Eur J Case Rep Intern Med. 2021;8:002870. 34790631 10.12890/2021_002870 PMC8592666

[10] Gorun F Tomescu L Motoc A. Clinical features and management of trophoblastic epithelioid tumors: a systematic review. Medicine (Baltimore). 2022;101:e29934. 35905248 10.1097/MD.0000000000029934 PMC9333520

[11] Frijstein MM Lok CAR van Trommel NE.; all the contributors to the ISSTD PSTT/ETT database. Management and prognostic factors of epithelioid trophoblastic tumors: results from the international society for the study of trophoblastic diseases database. Gynecol Oncol. 2019;152:361–7. 30473257 10.1016/j.ygyno.2018.11.015

[12] Gadducci A Carinelli S Guerrieri ME. Placental site trophoblastic tumor and epithelioid trophoblastic tumor: clinical and pathological features, prognostic variables and treatment strategy. Gynecol Oncol. 2019;153:684–93. 31047719 10.1016/j.ygyno.2019.03.011

[13] Helbig M Steinmann M Jaschinski S. Primary hepatic metastatic epithelioid trophoblastic tumor of the uterus treated with multimodal therapy including pembrolizumab and thermoablation. Case report of an extremely rare disease and review of the literature. Gynecol Oncol Rep. 2023;49:101281. 37822711 10.1016/j.gore.2023.101281 PMC10562736

[14] Chohan MO Rehman T Cerilli LA. Metastatic epithelioid trophoblastic tumor involving the spine. Spine (Phila Pa 1976). 2010;35:E1072–5. 20802395 10.1097/BRS.0b013e3181d7696b

[15] Ohira S Yamazaki T Hatano H. Epithelioid trophoblastic tumor metastatic to the vagina: an immunohistochemical and ultrastructural study. Int J Gynecol Pathol. 2000;19:381–6. 11109170 10.1097/00004347-200010000-00015

[16] Nakamura B Cowan M Griffin BB. Successful management of stage IV epithelioid trophoblastic tumor using multimodality treatment: a case report. Gynecol Oncol Rep. 2021;37:100802. 34195329 10.1016/j.gore.2021.100802 PMC8239721

[17] Xing D Zhong M Ye F. Ovarian intermediate trophoblastic tumors: genotyping defines a distinct category of nongestational tumors of germ cell type. Am J Surg Pathol. 2020;44:516–25. 31688005 10.1097/PAS.0000000000001402 PMC7373165

[18] Tsai HW Lin CP Chou CY. Placental site nodule transformed into a malignant epithelioid trophoblastic tumour with pelvic lymph node and lung metastasis. Histopathology. 2008;53:601–4. 18983471 10.1111/j.1365-2559.2008.03145.x

[19] Shih IM Kurman RJ. Epithelioid trophoblastic tumor: a neoplasm distinct from choriocarcinoma and placental site trophoblastic tumor simulating carcinoma. Am J Surg Pathol. 1998;22:1393–403. 9808132 10.1097/00000478-199811000-00010

[20] Papadopoulos AJ Foskett M Seckl MJ. Twenty-five years’ clinical experience with placental site trophoblastic tumors. J Reprod Med. 2002;47:460–4. 12092014

[21] Oldt RJ 3rd Kurman RJ Shih Ie M. Molecular genetic analysis of placental site trophoblastic tumors and epithelioid trophoblastic tumors confirms their trophoblastic origin. Am J Pathol. 2002;161:1033–7. 12213732 10.1016/s0002-9440(10)64264-2 PMC1867236

[22] Zhang X Shi H Chen X. Epithelioid trophoblastic tumor after induced abortion with previous broad choriocarcinoma: a case report and review of literature. Int J Clin Exp Pathol. 2014;7:8245–50. 25550880 PMC4270619

[23] Yigit S Gun E Yilmaz B. Epithelioid trophoblastic tumor in a postmenopausal woman: a case report and review of the literature in the postmenopausal group. Indian J Pathol Microbiol. 2020;63(Supplement):S98–S101. 32108639 10.4103/IJPM.IJPM_656_18

[24] Coulson LE Kong CS Zaloudek C. Epithelioid trophoblastic tumor of the uterus in a postmenopausal woman: a case report and review of the literature. Am J Surg Pathol. 2000;24:1558–62. 11075860 10.1097/00000478-200011000-00014

[25] Allan RW Algood CB Shih Ie M. Metastatic epithelioid trophoblastic tumor in a male patient with mixed germ-cell tumor of the testis. Am J Surg Pathol. 2009;33:1902–5. 19898219 10.1097/PAS.0b013e3181c03792

[26] Sakhadeo U Menon S Prakash G. Metastatic epithelioid trophoblastic tumor in retroperitoneal nodes in a case of regressed germ cell tumor of testis: an extremely rare occurrence. Indian J Urol. 2022;38:230–3. 35983109 10.4103/iju.iju_58_22 PMC9380464

[27] McGregor SM Furtado LV Montag AG. Epithelioid trophoblastic tumor: expanding the clinicopathologic spectrum of a rare malignancy. Int J Gynecol Pathol. 2020;39:8–18. 30480644 10.1097/PGP.0000000000000563

[28] Lewin SN Aghajanian C Moreira AL. Extrauterine epithelioid trophoblastic tumors presenting as primary lung carcinomas: morphologic and immunohistochemical features to resolve a diagnostic dilemma. Am J Surg Pathol. 2009;33:1809–14. 19773636 10.1097/PAS.0b013e3181b9cd67

[29] Kong Y Tao G Zong L. Diagnosis and management of mixed gestational trophoblastic neoplasia: a study of 16 cases and a review of the literature. Front Oncol. 2019;9:1262. 31803628 10.3389/fonc.2019.01262 PMC6873612

[30] Froeling FEM Ramaswami R Papanastasopoulos P. Intensified therapies improve survival and identification of novel prognostic factors for placental-site and epithelioid trophoblastic tumours. Br J Cancer. 2019;120:587–94. 30792530 10.1038/s41416-019-0402-0 PMC6461960

[31] Gallardo J Hummel K Siatecka H. Epithelioid trophoblastic tumor presenting as an adnexal mass: report of a diagnostically challenging case. Int J Surg Pathol. 2023;31:651–5. 35946122 10.1177/10668969221117983

[32] Mao TL Seidman JD Kurman RJ. Cyclin E and p16 immunoreactivity in epithelioid trophoblastic tumor--an aid in differential diagnosis. Am J Surg Pathol. 2006;30:1105–10. 16931955 10.1097/01.pas.0000209854.28282.87

[33] Wang YN Dong Y Wang L. Special epithelioid trophoblastic tumor: a case report. World J Clin Cases. 2022;10:9354–60. 36159420 10.12998/wjcc.v10.i26.9354 PMC9477662

[34] Xu ML Yang B Carcangiu ML. Epithelioid trophoblastic tumor: comparative genomic hybridization and diagnostic DNA genotyping. Mod Pathol. 2009;22:232–8. 18820674 10.1038/modpathol.2008.165

[35] Zhang X Zhou C Yu M. Coexisting epithelioid trophoblastic tumor and placental site trophoblastic tumor of the uterus following a term pregnancy: report of a case and review of literature. Int J Clin Exp Pathol. 2015;8:7254–9. 26261623 PMC4525957

[36] Shih IM Kurman RJ. p63 expression is useful in the distinction of epithelioid trophoblastic and placental site trophoblastic tumors by profiling trophoblastic subpopulations. Am J Surg Pathol. 2004;28:1177–83. 15316317 10.1097/01.pas.0000130325.66448.a1

[37] Stichelbout M Devisme L Franquet-Ansart H. SALL4 expression in gestational trophoblastic tumors: a useful tool to distinguish choriocarcinoma from placental site trophoblastic tumor and epithelioid trophoblastic tumor. Hum Pathol. 2016;54:121–6. 27068524 10.1016/j.humpath.2016.03.012

[38] Imamura Y Tashiro H Saito F. Choriocarcinoma coexisting with epithelioid trophoblastic tumor of the uterine horn. Gynecol Oncol Rep. 2015;14:31–3. 26793769 10.1016/j.gore.2015.10.002 PMC4688881

[39] Lukinovic N Malovrh EP Takac I. Advances in diagnostics and management of gestational trophoblastic disease. Radiol Oncol. 2022;56:430–9. 36286620 10.2478/raon-2022-0038 PMC9784364

[40] Ngu SF Ngan HYS. Surgery including fertility-sparing treatment of GTD. Best Pract Res Clin Obstet Gynaecol. 2021;74:97–108. 33127305 10.1016/j.bpobgyn.2020.10.005 PMC7547826

[41] Schmid P Nagai Y Agarwal R. Prognostic markers and long-term outcome of placental-site trophoblastic tumours: a retrospective observational study. Lancet. 2009;374:48–55. 19552948 10.1016/S0140-6736(09)60618-8

[42] Yang J Zong L Wang J. Epithelioid trophoblastic tumors: treatments, outcomes, and potential therapeutic targets. J Cancer. 2019;10:11–9. 30662520 10.7150/jca.28134 PMC6329873

[43] Zhang T Zeng X Xu H. Clinical characteristics and outcomes of extrauterine epithelioid trophoblastic tumors. Arch Gynecol Obstet. 2019;300:725–35. 31312959 10.1007/s00404-019-05239-0

[44] Sugrue R Foley O Elias KM. Outcomes of minimally invasive versus open abdominal hysterectomy in patients with gestational trophoblastic disease. Gynecol Oncol. 2021;160:445–9. 33272644 10.1016/j.ygyno.2020.11.022

[45] Abu-Rustum NR Yashar CM Bean S. Gestational trophoblastic neoplasia, version 2.2019, NCCN clinical practice guidelines in oncology. J Natl Compr Canc Netw. 2019;17:1374–91. 31693991 10.6004/jnccn.2019.0053

[46] Coopmans L Larsson A Joneborg U. Surgical management of gestational trophoblastic disease. Gynecol Obstet Invest. 2023. 10.1159/000534065 37788661

[47] Horowitz NS Goldstein DP Berkowitz RS. Placental site trophoblastic tumors and epithelioid trophoblastic tumors: biology, natural history, and treatment modalities. Gynecol Oncol. 2017;144:208–14. 27789086 10.1016/j.ygyno.2016.10.024

[48] Burkett WC Soper JT. A review of current management of placental site trophoblastic tumor and epithelioid trophoblastic tumor. Obstet Gynecol Surv. 2022;77:101–10. 35201361 10.1097/OGX.0000000000000978

[49] Liu W Zhou J Yang J. A multicenter retrospective study of epithelioid trophoblastic tumors to identify the outcomes, prognostic factors, and therapeutic strategies. Front Oncol. 2022;12:907045. 35677151 10.3389/fonc.2022.907045 PMC9169038

[50] Sobecki-Rausch J Winder A Maniar KP. Surgery and platinum/etoposide-based chemotherapy for the treatment of epithelioid trophoblastic tumor. Int J Gynecol Cancer. 2018;28:1117–22. 29757875 10.1097/IGC.0000000000001278

[51] Zhang X Lu W Lu B. Epithelioid trophoblastic tumor: an outcome-based literature review of 78 reported cases. Int J Gynecol Cancer. 2013;23:1334–8. 23970158 10.1097/IGC.0b013e31829ea023

[52] Clark JJ Slater S Seckl MJ. Treatment of gestational trophoblastic disease in the 2020s. Curr Opin Obstet Gynecol. 2021;33:7–12. 33337613 10.1097/GCO.0000000000000674 PMC7116872

[53] Hou YM Li PP Yu H. Clinical features and demographic characteristics of gestational trophoblastic neoplasia: Single center experience and the SEER database. Biomol Biomed. 2024;24:176–87. 37485958 10.17305/bb.2023.9092 PMC10787625

[54] Zhang Y Zhang S Huang W. Intermediate trophoblastic tumor: the clinical analysis of 62 cases and prognostic factors. Arch Gynecol Obstet. 2019;299:1353–64. 30607597 10.1007/s00404-018-05037-0

[55] Shen X Xiang Y Guo L. Analysis of clinicopathologic prognostic factors in 9 patients with epithelioid trophoblastic tumor. Int J Gynecol Cancer. 2011;21:1124–30. 21738043 10.1097/IGC.0b013e31821dc89a

[56] Scott EM Smith AL Desouki MM. Epithelioid trophoblastic tumor: a case report and review of the literature. Case Rep Obstet Gynecol. 2012;2012:862472. 23243530 10.1155/2012/862472 PMC3518084

[57] Palmer JE Macdonald M Wells M. Epithelioid trophoblastic tumor: a review of the literature. J Reprod Med. 2008;53:465–75. 18720920

[58] Cheng H Zong L Yu S. Expression of the immune targets in tumor-infiltrating immunocytes of gestational trophoblastic neoplasia. Pathol Oncol Res. 2023;29:1610918. 36875956 10.3389/pore.2023.1610918 PMC9977799

[59] Fenichel P Rouzier C Butori C. Extragestational betaHCG secretion due to an isolated lung epithelioid trophoblastic tumor: microsatellite genotyping of tumoral cells confirmed their placental origin and oriented specific chemotherapy. J Clin Endocrinol Metab. 2014;99:3515–20. 25029419 10.1210/jc.2014-1460

[60] Li J Shi Y Wan X. Epithelioid trophoblastic tumor: a clinicopathological and immunohistochemical study of seven cases. Med Oncol. 2011;28:294–9. 20087692 10.1007/s12032-010-9419-1

[61] Black L Bowes A Seckl M. Epithelioid trophoblastic tumor with antecedent molar pregnancy in an HIV-positive patient. Clin Case Rep. 2023;11:e7114. 36998325 10.1002/ccr3.7114 PMC10043136

[62] Urabe S Fujiwara H Miyoshi H. Epithelioid trophoblastic tumor of the lung. J Obstet Gynaecol Res. 2007;33:397–401. 17578376 10.1111/j.1447-0756.2007.00545.x

[63] Niu N Ordulu Z Burak Z. Extrauterine epithelioid trophoblastic tumour and its somatic carcinoma mimics: short tandem repeat genotyping meets the diagnostic challenges. Histopathology. 2024;84:325–35. 37743102 10.1111/his.15054

[64] Knox S Brooks SE Wong-You-Cheong J. Choriocarcinoma and epithelial trophoblastic tumor: successful treatment of relapse with hysterectomy and high-dose chemotherapy with peripheral stem cell support: a case report. Gynecol Oncol. 2002;85:204–8. 11925147 10.1006/gyno.2002.6583

[65] Ahn HY Hoseok I Lee CH. Pulmonary mass diagnosed as extrauterine epithelioid trophoblastic tumor. Thorac Cardiovasc Surg. 2013;61:97–100. 23307273 10.1055/s-0032-1331264

[66] Okereke IC Chen S. Primary epithelioid trophoblastic tumor of the lung. Ann Thorac Surg. 2014;97:1420–1. 24694417 10.1016/j.athoracsur.2013.07.031

[67] Abrao FC Sabbion RO Canzian M. Isolated epithelioid trophoblastic tumor mimicking non-small cell lung cancer. J Thorac Oncol. 2011;6:966–7. 21623270 10.1097/JTO.0b013e318215a214

[68] Kim JY An S Jang SJ. Extrauterine epithelioid trophoblastic tumor of lung in a 35-year-old woman. Korean J Thorac Cardiovasc Surg. 2013;46:471–4. 24368977 10.5090/kjtcs.2013.46.6.471 PMC3868698

[69] Li JW Hu CC Shi HY. Extrauterine epithelioid trophoblastic tumors presenting as lung mass: a case report and literature review. Medicine (Baltimore). 2019;98:e14010. 30702558 10.1097/MD.0000000000014010 PMC6380824

[70] Fadare O Parkash V Carcangiu ML. Epithelioid trophoblastic tumor: clinicopathological features with an emphasis on uterine cervical involvement. Mod Pathol. 2006;19:75–82. 16258513 10.1038/modpathol.3800485

[71] Jashnani K Yagana A Mahajan N. Double trouble: extrauterine epithelioid trophoblastic tumor with uterine choriocarcinoma – an autopsy report. Indian J Cancer. 2020;57:463–6. 33078754 10.4103/ijc.IJC_220_19

[72] Kuo KT Chen MJ Lin MC. Epithelioid trophoblastic tumor of the broad ligament: a case report and review of the literature. Am J Surg Pathol. 2004;28:405–9. 15104307 10.1097/00000478-200403000-00017

[73] Zeng C Rezai S Hughes AC. Synchronous choriocarcinoma and epithelioid trophoblastic tumor concurring at the cesarean scar: a case report and review of the literature. Case Rep Obstet Gynecol. 2019;2019:5093938. 31637071 10.1155/2019/5093938 PMC6766115

[74] Hsiue EH Hsu C Tseng LH. Epithelioid trophoblastic tumor around an abdominal cesarean scar: a pathologic and molecular genetic analysis. Int J Gynecol Pathol. 2017;36:562–7. 28134666 10.1097/PGP.0000000000000366

[75] Yang C Li J Zhang Y. Epithelioid trophoblastic tumor coexisting with choriocarcinoma around an abdominal wall cesarean scar: a case report and review of the literature. J Med Case Rep. 2020;14:178. 33012293 10.1186/s13256-020-02485-8 PMC7534162

[76] Khunamornpong S Settakorn J Sukpan K. Ovarian involvement of epithelioid trophoblastic tumor: a case report. Int J Gynecol Pathol. 2011;30:167–72. 21293282 10.1097/PGP.0b013e3181f7124e

[77] Park SY Park MH Ko HS. Epithelioid trophoblastic tumor presenting as an ovarian mass in a postmenopausal woman. Int J Gynecol Pathol. 2014;33:35–9. 24300533 10.1097/PGP.0000000000000074

[78] Mahmood H Faheem M Tahir M. Epithelioid trophoblastic tumor: an unusual malignancy of ovary. J Coll Physicians Surg Pak. 2014;24(Suppl 3):S201–3. 25518773

[79] Zhao J Xiang Y Zhao D. Isolated epithelioid trophoblastic tumor of the vagina: a case report and review of the literature. Onco Targets Ther. 2013;6:1523–6. 24194644 10.2147/OTT.S50553 PMC3814932

[80] Keser SH Kokten SC Cakir C. Epithelioid trophoblastic tumor. Taiwan J Obstet Gynecol. 2015;54:621–4. 26522123 10.1016/j.tjog.2015.08.020

[81] Theodossiadis P Rouvas A Nakopoulou L. Epithelioid trophoblastic tumor. Ophthalmology. 2007;114:1421. 17613344 10.1016/j.ophtha.2007.04.002

[82] Macdonald MC Palmer JE Hancock BW. Diagnostic challenges in extrauterine epithelioid trophoblastic tumours: a report of two cases. Gynecol Oncol. 2008;108:452–4. 18078982 10.1016/j.ygyno.2007.11.015

[83] Madhu B Gerbi R Nabila R. Epithelioid trophoblastic tumor and its diagnostic dilemmas: a rare case report. Gynecol Oncol Case Rep. 2011;2:42–3. 24371611 10.1016/j.gynor.2011.12.003 PMC3861130

[84] Bell SG Uppal S Sakala MD. An extrauterine extensively metastatic epithelioid trophoblastic tumor responsive to pembrolizumab. Gynecol Oncol Rep. 2021;37:100819. 34258359 10.1016/j.gore.2021.100819 PMC8258853

[85] Aiob A Cohen HI Naskovica K. Coexisting epithelioid trophoblastic tumor and placental site trophoblastic tumor during asymptomatic relapse: a case report and literature review. Int J Gynecol Pathol. 2022;41:423–30. 34392267 10.1097/PGP.0000000000000810

[86] Tse KY Chiu KWH Chan KKL. A case series of five patients with pure or mixed gestational epithelioid trophoblastic tumors and a literature review on mixed tumors. Am J Clin Pathol. 2018;150:318–32. 29897391 10.1093/ajcp/aqy039

[87] Jiang F Xiang Y Guo LN. Laparoscopic diagnosis and treatment of an isolated epithelioid trophoblastic tumor in recto-uterine pouch. J Obstet Gynaecol Res. 2018;44:960–5. 29436119 10.1111/jog.13593

[88] Gil F Elvas L Raposo S. Keratoacanthoma-like nodules as first manifestation of metastatic epithelioid trophoblastic tumor. Dermatol Online J. 2019;25:13030/qt9xx6p2tt. 31735008

[89] Parker A Lee V Dalrymple C. Epithelioid trophoblastic tumour: report of a case in the fallopian tube. Pathology (Phila). 2003;35:136–40. 12745461

[90] Noh HT Lee KH Lee MA. Epithelioid trophoblastic tumor of paracervix and parametrium. Int J Gynecol Cancer. 2008;18:843–6. 17944924 10.1111/j.1525-1438.2007.01086.x

[91] Frijstein MM Lok CAR Short D. The results of treatment with high-dose chemotherapy and peripheral blood stem cell support for gestational trophoblastic neoplasia. Eur J Cancer. 2019;109:162–71. 30731277 10.1016/j.ejca.2018.12.033

[92] El-Helw LM Seckl MJ Haynes R. High-dose chemotherapy and peripheral blood stem cell support in refractory gestational trophoblastic neoplasia. Br J Cancer. 2005;93:620–1. 16222307 10.1038/sj.bjc.6602771 PMC2361618

[93] Ghorani E Kaur B Fisher RA. Pembrolizumab is effective for drug-resistant gestational trophoblastic neoplasia. Lancet. 2017;390:2343–5. 29185430 10.1016/S0140-6736(17)32894-5

[94] Qian XQ Shen YM Wan XY. Epithelioid trophoblastic tumor that requires fertility preservation: a case report and review of literature. Taiwan J Obstet Gynecol. 2020;59:736–9. 32917327 10.1016/j.tjog.2020.07.019

